# The Influence of Missing Data on Disabilities in Patients Treated with High-Dose Spinal Cord Stimulation: A Tipping Point Sensitivity Analysis

**DOI:** 10.3390/jcm10214897

**Published:** 2021-10-24

**Authors:** Lisa Goudman, Geert Molenberghs, Rui V. Duarte, Maarten Moens

**Affiliations:** 1 Department of Neurosurgery, Universitair Ziekenhuis Brussel, Laarbeeklaan 101, 1090 Brussels, Belgium; maarten.moens@uzbrussel.be; 2 STIMULUS Research Group (reSearch and TeachIng neuroModULation Uz bruSsel), Vrije Universiteit Brussel, Laarbeeklaan 103, 1090 Brussels, Belgium; 3 Center for Neurosciences (C4N), Vrije Universiteit Brussel, Laarbeeklaan 103, 1090 Brussels, Belgium; 4 Pain in Motion International Research Group, Laarbeeklaan 103, 1090 Brussels, Belgium; 5 Research Foundation—Flanders (FWO), 1090 Brussels, Belgium; 6 Interuniversity Institute for Biostatistics and Statistical Bioinformatics (I-BIOSTAT), Data Science Institute, Hasselt University, 3590 Diepenbeek, Belgium; geert.molenberghs@uhasselt.be; 7 Interuniversity Institute for Biostatistics and Statistical Bioinformatics (I-BIOSTAT), Catholic University of Leuven, 3000 Leuven, Belgium; 8 Liverpool Reviews and Implementation Group, Department of Health Data Science, University of Liverpool, Liverpool L69 3BX, UK; rui.duarte@liverpool.ac.uk; 9 Department of Radiology, Universitair Ziekenhuis Brussel, Laarbeeklaan 101, 1090 Brussels, Belgium

**Keywords:** missing data mechanisms, sensitivity analysis, multiple imputations, neuromodulation

## Abstract

New waveforms have changed the field of Spinal Cord Stimulation (SCS) to optimize therapy outcomes, among which is High-Dose SCS (HD-SCS). Missing observations are often encountered when conducting clinical trials in this field. In this study, different approaches with varying assumptions were constructed to evaluate how conclusions may be influenced by these assumptions. The aim is to perform a tipping point sensitivity analysis to evaluate the influence of missing data on the overall conclusion regarding the effectiveness of HD-SCS on disability. Data from the Discover study were used, in which 185 patients with Failed Back Surgery Syndrome were included. Disability was evaluated before SCS and after 1, 3 and 12 months of HD-SCS. During the second, third and fourth visit, data from 130, 114 and 90 patients were available, respectively. HD-SCS resulted in a significant decrease in disability scores based on the analysis of observed data and with multiple imputations. The tipping point sensitivity analysis revealed that the shift parameter was 17. Thus, the conclusion concerning the time effect under a “missing at random” mechanism is robust when the shift parameter for the disability score is 17. From a clinical point of view, a shift of 17 points on disability is not very plausible. Therefore we tend to consider the conclusions drawn under “missing at random” as being robust.

## 1. Introduction

Spinal Cord Stimulation (SCS) has been established as an effective therapy to treat a wide variety of chronic pain conditions among which patients with Failed Back Surgery Syndrome [1], recently called Persistent Spinal Pain Syndrome Type II (PSPS T2). This condition is characterized by persistent back and/or leg pain of unknown origin either despite the surgical intervention or appearing after surgical intervention for spinal pain [2]. The goal of SCS is not to cure patients but rather to make chronic pain tolerable, with benefits on function and health-related quality of life [3,4].

Initially, conventional, low-frequency SCS was provided whereby patients experience paresthesia in the painful areas [5,6]. Over the last decade, several new waveforms and frequencies were introduced that do not induce paresthesia [6,7]. One of those new paresthesia-free stimulation paradigms is High-Dose SCS (HD-SCS), formerly known as high-density SCS [8]. HD-SCS entails an increase in frequency and pulse width, along with reduced amplitude, when compared to standard SCS [7]. Despite the absence of an exact definition for the stimulation parameters of HD-SCS, the delivery of energy to neural tissue is the key concept [9]. The percentage of active stimulation during a pulse cycle can be increased up to 20–25% for the maximally available settings, at a sub sensory mode [6,7].

When evaluating the success of treatment in the field of neuromodulation, the most prominent outcome measurement is a reduction in pain intensity [10]. However, it has previously been demonstrated that achieving a pre-treatment goal of “reducing pain” contributes very little to patient satisfaction in the chronic disabled back and/or neck pain patients [11]. Moreover, achieving functional goals was more important for patient satisfaction than a reduction in self-reported pain [11]. Additionally, a qualitative exploration of patients’ expectations on SCS indicated that patients have more expectations than only obtaining pain relief [12]. These studies, combined with the recent calls in the SCS literature to focus on a combination of several outcome measurements [13,14,15,16], clearly demonstrate that we should redefine the concept of successful treatment in SCS. Disability is one of the factors that can be proposed as an additional self-reporting measure for evaluating the treatment effects of SCS [15,17]. One of the most frequently used questionnaires to evaluate disability within patients with chronic low back pain is the Oswestry Disability Index (ODI) [18]. This questionnaire, initially developed by O’Brien in 1976, consists of ten sections measuring pain intensity, personal care, lifting, walking, sitting, standing, sleeping, sex life, social life, and traveling [18].

Missing observations are one of the most common but often overlooked issues that are encountered when conducting clinical trials [19]. In 1987, Little and Rubin classified the missing data mechanisms in three distinct categories namely missing completely at random (MCAR; the observed responses can be seen as a random subsample of the sampled responses, given covariates), missing at random (MAR; the probability of missingness depends on the observed data but given these not further on the unobserved data) and missing not at random (MNAR) [20]. A sensible starting point for clinical trials is the MAR assumption; however, the exact missingness mechanism cannot be formally evaluated [21]. It thus becomes clear that performing analyses on incomplete data requires untestable assumptions, pointing out the necessity of understanding the impact of these assumptions on inferences and conclusions from the primary analysis. Sensitivity analysis entails the creation of different models with varying assumptions and evaluating how conclusions are influenced [22]. The aim of this study is to perform a sensitivity analysis on disability data to evaluate the influence of missingness on the overall conclusion regarding the effectiveness of HD-SCS on disability in patients with PSPS T2.

## 2. Materials and Methods

### 2.1. Data

Data from the Discover study, a prospective multicenter registry-based cohort study towards the effectiveness of HD-SCS, were used in this study. The protocol was prospectively registered at clinicaltrials.gov (NCT02787265) on 1 June 2016. The protocol of this study and the main results can be found elsewhere [15,23]. Patients with PSPS T2, and a numerical rating scale (NRS) score ≥5/10 for leg and/or back pain were eligible. All patients underwent a baseline visit before lead implantation. Subsequently, patients underwent a trial period of 4 weeks with an external neurostimulator. In case of a successful trial, in which an average pain reduction of 50% and a reduction in pain medication use of at least 50% should be obtained, an internal pulse generator (IPG) was implanted. All patients were implanted with a RestoreSensor, Intellis or PrimeAdanced IPG (Minneapolis, MN, USA) and received HD-SCS with a pulse density of 25% (500 Hz and 500 sec of pulse) in case of the RestoreSensor or Intellis and 11.7% (450 Hz and 130 sec of pulse width) in case of a PrimeAdvanced IPG. Finally, 3 follow-up visits took place after 1, 3, and 12 months of HD-SCS.

The study was conducted according to the revised Declaration of Helsinki (1998). The study protocol was approved by the ethics committee of Universitair Ziekenhuis Brussel (B.U.N. 143201629180) and the ethics committees of each participating center. All patients provided written informed consent before enrolment in this study.

### 2.2. Outcome Measurements

The primary outcome measurement for this analysis was the ODI, consisting of ten items describing functional aspects of daily living. Each item contains six statements with a total score of 5 [24]. A total score of 100% indicates total disability. In PSPS T2, the ODI has a sensitivity of 0.74 and a specificity of 0.92 [25].

Current pain intensity was measured with an NRS, ranging from 0 (no pain) to 10 (maximal pain) for low back pain and leg pain separately.

### 2.3. Statistical Analysis

Due to the repeated measures nature of the data, longitudinal mixed models were used to evaluate the effectiveness of HD-SCS on disability in patients with PSPS T2. Concerning model building, a nearly saturated mean model was fitted that included all main effects, two-way and three-way interaction terms. Age (young patients (25–45 years), middle-aged patients (46–65 years) and older patients (66–85 years)), sex, follow-up visit (time), NRS low back pain at baseline and NRS leg pain at baseline were used as predictors. Firstly, a model with an unstructured covariance matrix was constructed, allowing different variances at each visit. The necessity of random slopes and/or random intercepts was evaluated by Restricted Maximum Likelihood (REML) estimation. Splines were also considered (REML estimation). Secondly, an unstructured covariance matrix was compared with other covariance structures. Model selection was performed with Akaike information criterion (AIC) values. If the unstructured covariance matrix model did not differ significantly from a model with more restrictive assumptions, the unstructured model was replaced. Thirdly, the fixed part of the model was simplified by dropping unnecessary predictors using likelihood ratio tests, starting from the interaction terms. Deletion of a predictor was allowed if it did not affect the model (*p* > 0.05). If a higher-order interaction term needed to be included, the lower interaction terms and main effects remained in the model as well. All statistical analyses were performed in SAS 9.4 (100 SAS Campus Drive Cary, NC, USA) with PROC MIXED.

### 2.4. Sensitivity Analysis

A wide variety of methods for handling missing data are available, whereby imputation methods are widely applied in which the missing observation is filled up with a plausible value [26]. A commonly used technique is the “last observation carried forward” method whereby the missing value is imputed with the last available observation [27]. The disadvantage of such single imputation methods is that they do not account for uncertainty, thereby provoking an underestimation of the standard error of the statistical point estimates [28]. In addition, they are prone to sometimes severe biases. A second and quite different type of imputation is multiple imputations in which the main idea is to replace every missing value by a set of M ≥ 2 plausible values [29]. The vector of M values is constructed based on repeated draws from the posterior predictive distribution of the unobserved values given the observed ones [30]. This implies that we assume MAR during multiple imputations because the predictive distribution of the missing data, given the observed data, does not depend on the observed response [31]. These M values represent the uncertainty about the value, in contrast to simple imputation strategies. By the end of this step, all missing values are filled in with M values to generate M complete datasets. Standard methods are applied to analyze each dataset separately where after M inferences are then combined to withhold one inference that is properly reflecting the sampling variability due to missing values under the considered model [30]. Although multiple imputations assume MAR, the exact missing mechanism cannot be formally evaluated. In a tipping point analysis, the influence of missingness is explored on the overall conclusion from the statistical inference by applying a wide spectrum of different assumptions regarding the missingness mechanisms [28]. The aim is to find the “tipping point” in the spectrum of assumptions at which conclusions from the statistical inference will be changed [28]. Afterward, a clinical interpretation can be given to the plausibility of the assumptions [32]. If the value of the shift parameter that changes the conclusions of the main study inference is implausible, then greater confidence can be inferred from the main results [29]. A tipping point that is situated at a biologically or clinically implausible location provides evidence for the robustness of the conclusions reached under MAR.

Firstly, a dataset without missingness was created using multiple imputation strategies. Two approaches were consecutively performed: (1) creation of a dataset with only monotone missingness; (2) creation of a dataset without missingness by regression-based imputation (PROC MI procedure). The former is achieved with a Markov Chain Monte Carlo method using a multivariate normal model [33]. The advantage of the latter is that a sequential approach with univariate models with a number of predictor variables is used. This enables first imputing data from the earliest visit, whereby the outcome can then be used as predictor for imputations at later visits [34]. The imputation model included the previous outcomes of the dependent variable combined with covariates age and pain intensity scores. Ten imputations were created for each missing value.

Secondly, a set of shift parameters with a progressive increased departures from MAR (shift = 0) is applied. A wide spectrum of shifts for the values that were missing was assumed, ranging from a decrease of −30 on the ODI up to + 30. Next, the same steps as above were conducted to generate multiply imputed datasets, with a specified shift parameter that adjusts the imputed values. Thereafter, the imputed datasets were analyzed by using the same likelihood analysis as in the primary analysis (PROC MIXED procedure). Inferences were then combined for each shift parameter until a *p*-value of 0.05 or higher was revealed for the main study inference (MI ANALYZE procedure). The basis for this analysis was the SAS macro by Yang (2013) for RCT’s, available from the SAS help center.

## 3. Results

### 3.1. Descriptive Statistics

In this study, 89 males (48.1%) and 96 females (51.9%) were included with a mean age of 54 (SD 12.01) years. The mean ODI score at baseline was 56.99 (SD 14.97), 31.26 (SD 17.58) at 1 month, 30.64 (SD 18.52) at 3 months and 33.34 (SD 16.86) at 12 months. At the baseline visit, data of 185 patients were available for the ODI. During the second, third and fourth visit, data of 130, 114 and 90 patients were available, respectively.

### 3.2. Longitudinal Effect of HD-SCS on Disability

**Figure 1:**
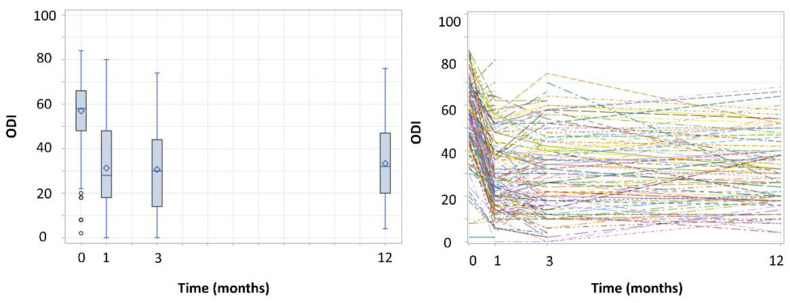
Distribution of total score on the Oswestry Disability Index (ODI) per study visit, presented with boxplots (**left**) and individual profile plots (**right**). The mean is represented by the diamond, the horizontal line represents the median ODI score at each visit.

Overall, a decrease in average ODI scores over time was observed (Figure 1).

There seemed to be lower variability in ODI score at baseline compared to the follow-up visits. The ODI score seemed to decrease very fast from baseline to one month of SCS, wherefore a linear spline was added to the model. The main idea behind this concept is to divide the time axis into a series of segments and consider a model for the trend over time that is comprised of piecewise linear trends, with different slopes within each segment that are tied together at fixed times (denoted as “knots”) [31]. Therefore, a knot was assumed at the first month’s visit thanks to the creation of an additional variable “Time1” which took the value zero when the observation occurred before the first month. Otherwise, the new variable took the value of the current month minus one.

The regression coefficient estimates of the final model are presented in Table 1.

At the baseline visit, the average ODI score is 25.05 ((95% CI: 20.43–29.66), *p* ≤ 0.001) for a patient with a pain intensity score of 0 for both back and leg pain. For 95% of the patients, the average ODI score before treatment (i.e., at baseline) varies between 4.83 and 45.27. Per unit increase in NRS low back pain score, the average ODI score will increase with 2.32 ((95% CI: 1.82–2.81), F = 86.73, *p* ≤ 0.001). For each unit increase in NRS leg pain score, the average ODI score will increase by 1.87 units ((95% CI: 1.44–2.30), F = 75.38, *p* ≤ 0.001). During the first month of HD-SCS, there is an average decrease of 7.68 ((95% CI: 4.98–10.39), F = 31.58, *p* ≤ 0.001) points in the ODI score. The percentage of patients that are experiencing an average decrease in ODI score during the first month of HD-SCS is 84.7%. From 1 month of HD-SCS onwards, an increase of 7.61 ((95% CI: 4.84–10.38), F = 29.66, *p* ≤ 0.001) in ODI score is revealed per visit. The autoregressive correlation parameter of 0.6639 indicated that, for a middle-aged patient, the correlation between two visits that were one time unit apart was 0.6639. The correlation between older patients and young patients was 0.18 and 0.35, respectively, between two visits that were one time unit apart.

### 3.3. Sensitivity Analysis

Based on the previous analysis, HD-SCS was able to significantly decrease disability scores over time in patients with PSPS T2, based on an analysis of data as observed. However, a substantial proportion of the data was missing. Table 2 provides an overview of the different types of missing data.

In total, 43.78% of the patients were compliant with all visits, 50.82% exhibited monotone missingness and 5.4% exhibited non-monotone missingness (Table 2). Within the group with monotone missingness, a considerable amount of patients has no follow-up measurements (25.41%), 9.19% have one follow-up visit and 16.22% have two follow-up visits.

In Table 3, the main effects of the primary analysis under multiple imputations are presented. Type III tests for time and time1 remained statistically significant in the analysis with multiple imputations, as observed in the analysis without imputation.

In Table 4, the results of the tipping point analysis are presented.

**Figure 2:**
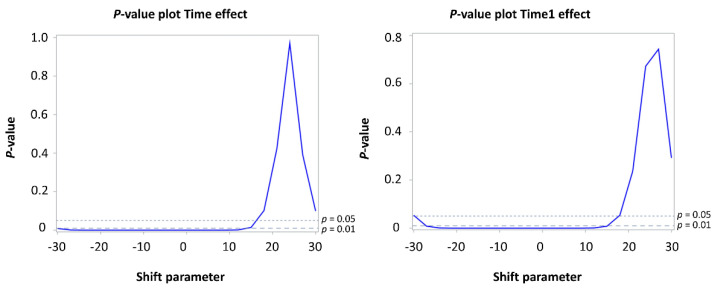
Tipping point analysis for shift parameters ranging from −30 to 30 for time effect (**left**) and time1 effect (**right**).

A shift of zero corresponds to a standard MAR-based multiple imputation analysis. When the ODI is shifted with a value of slightly less than 18 (meaning patients with missing values experience more disability), the conclusion becomes different from the likelihood-based analysis. More specifically, for a two-sided error level of 0.05, the tipping point for the shift parameter is 17 for the time effect and 18 for the time1 effect. Thus, the study conclusion under MAR is reversed when the shift parameter is 17. This means that if the shift parameter of 17 is plausible, the conclusion under MAR is questionable. Visually, the results are presented in Figure 2.

## 4. Discussion

The effectiveness outcome in this analysis was defined as a mean disability reduction over time following HD-SCS as measured by the ODI. Based on a longitudinal mixed model, the mean disability reduction reached a strong statistical significance over time. The decrease in disability in PSPS T2 patients who are treated with HD-SCS was not surprising. In a study with multicolumn SCS, a significant decrease in ODI scores was found between baseline and 6 months of SCS [3]. In the SENZA RCT (comparison of 10 kHz SCS to conventional SCS for the treatment of chronic back and leg pain), the efficacy of 10 kHz SCS was explored whereby the ODI was measured as a secondary outcome variable. A significant change in ODI score was revealed after 12 months [35]. The final model in the current study contained a knot which indicated that there is a different slope from baseline to 1 month and from 1 month onwards. The difference in slope can be explained by habituation, since approximately 20–40% of SCS patients suffer from a decline in initial effectiveness of SCS due to a central nervous system tolerance, as already reported in 1993 by LeDoux [36]. This decline is often reported for pain relief [37]. However, in this study, this phenomenon was also observed for ODI. Drastic improvements in ODI scores were visible up to 1 month, where after a slight decrease became visible from 3 months onwards. This trend was also mentioned in a study with health-related quality of life after 6 months of SCS [38]. This suggests that habituation might be an issue in SCS in general but also on the level of disability, which could potentially have a major influence on the long-term clinical effects and therefore also in terms of salvage therapy and system explants [39].

Recently, recommendations on the levels of study design, site selection, participant selection, treatment adherence, data collection and data monitoring were created to improve the quality of clinical trials of chronic pain treatments [40]. In clinical trials with chronic pain patients, patient dropout is common and typically estimated at around 20% to 50% of trial participants, depending on the medication, dosage, pain condition, follow-up duration, and other factors [41,42]. During the last decades, more principled methods for handling missing data have been implemented such as ignorable likelihood, multiple imputations or weighted generalized estimating equations, whereby direct likelihood is expected to be the most frequently used technique in chronic pain trials [42]. With only missing data in the outcome, maximizing the likelihood of the observed data is expected to provide valid inference (given an appropriate choice for the covariance structure) [43]. With missing values in the dependent and independent variables, multiple imputations is often considered the most flexible and practical approach [43]. Nevertheless, these methods rely on strong and untestable assumptions concerning the conditional distribution of outcomes after dropout, given the observed data [42], wherefore sensitivity analysis should be conducted to explore whether the conclusions from the analysis under MAR are robust [43].

In the current multicenter registry, a rather large proportion of missing data was present wherefore a sensitivity analysis (assuming MNAR) was performed after the primary analysis, as recommended by the ICH (International Council for Harmonisation of Technical Requirements for Pharmaceuticals for Human Use) E9 guidance on Statistical Principles for Clinical Trials [44]. Both monotone and non-monotone missingness patterns were observed, whereby a two-step procedure was applied to impute missing data. More imputation techniques are available for monotone missingness [45], wherefore data were first imputed towards monotone missingness and then in a second step towards no missing data. From a clinical point of view, however, monotone missingness is a major issue since no information could be retrieved concerning the underlying reason for missing data. Only one possible sensitivity analysis was performed namely the tipping-point analysis. Within this type of analysis, it was explored how severe departures from MAR must be in order to reverse conclusions from the primary analysis. In this study, a shift parameter of 17 was needed to change the main conclusions of the longitudinal mixed model. A departure of 17 is rather large, moreover, it is well above the minimal clinical important difference (MCID) of the ODI which is estimated at 8–10 points [46,47,48]. In this study, the interpretation of the magnitude of the shift parameter was based on the MCID value, indicating that the shift parameter was almost twice the MCID value. Thus, from a clinical point of view, this parameter is not very plausible, indicating the robustness of the results of the previously performed statistical methods. Therefore, we can be more confident in the results obtained with statistical methods under the MAR assumptions namely the mixed model repeated measurements and multiple imputations; both pointing towards a significant time effect. Another approach to interpret the magnitude of the shift parameter is to determine a priori an acceptable range of assumptions for this specific study context with all trial team members. Caution is needed when tipping point approaches are performed without a clear rationale for the interpretation of the shift parameter since the results of the analysis might (un)knowingly influence the subsequent interpretation of the sensitivity analysis [29]. Given the lack of a universally determined best MNAR method [49], one should ideally explore a variety of sensitivity analysis in order to better evaluate the consistency/robustness of results across the various assumptions that are made with different techniques.

The Discover study was a longitudinal cohort study, exploring the effectiveness of HD-SCS in patients with PSPS T2. This study did not evaluate the efficacy of SCS compared to another treatment modality, which would have enabled us to differentiate the time effect from the treatment effect.

## 5. Conclusions

This is the first study to report longitudinal data on disability in patients with PSPS T2 who are treated with HD-SCS. In patients with PSPS T2, HD-SCS is an effective treatment option to decrease disability. Sensitivity analysis indicated that the results are maintained when the shift parameter is 17. From a clinical perspective, this shift does not seem very realistic therefore the conclusion under MAR can be considered as robust.

## Figures and Tables

**Table 1:** Regression coefficient estimates and their 95% confidence intervals, based on the final model.

Variable	Regression Estimates	Standard Error	95% Confidence Interval	Type III Test
Intercept	25.05	2.34	(20.43–29.66)	*p* < 0.001
NRS low back	2.32	0.25	(1.82–2.81)	*p* < 0.001
NRS leg	1.87	0.21	(1.44–2.30)	*p* < 0.001
Time	−7.68	1.37	(−10.39–−4.98)	*p* < 0.001
Time1	7.61	1.40	(4.84–10.38)	*p* < 0.001

**Table 2:** Overview of missingness patterns.

Type	Baseline	1 Month	3 Months	12 Months	Number	Percentage
Completers	O	O	O	O	81	43.78%
Monotone missingness	O	O	O	M	30	16.22%
	O	O	M	M	17	9.19%
	O	M	M	M	47	25.41%
Non-monotone missingsness	O	O	M	O	2	1.08%
	O	M	O	O	2	1.08%
	O	M	O	M	1	0.54%
	O	M	M	O	5	2.70%

Abbreviations. M: missing, O: observed.

**Table 3:** Regression coefficient estimates and their 95% confidence intervals, based on the final model with multiple imputations.

Variable	Regression Estimates	Standard Error	95% Confidence Interval	Type III Test
Intercept	25.65	2.26	(21.19–30.12)	*p* < 0.001
NRS low back	2.27	0.23	(1.81–2.73)	*p* < 0.001
NRS leg	1.83	0.20	(1.43–2.23)	*p* < 0.001
Time	−8.51	1.44	(−11.39–−5.63)	*p* < 0.001
Time1	8.46	1.49	(5.49–11.43)	*p* < 0.001

**Table 4:** Tipping point sensitivity analysis with *p*-values for time effects with shifts ranging from −30 to 30.

Shift	*p*-Value Time	*p*-Value Time1
−30	0.0087	0.0536
−27	0.0010	0.0082
−24	0.0001	0.0009
−21	<0.0001	0.0001
−18	<0.0001	<0.0001
−15	<0.0001	<0.0001
−12	<0.0001	<0.0001
−9	<0.0001	<0.0001
−6	<0.0001	<0.0001
−3	<0.0001	<0.0001
0	<0.0001	<0.0001
3	<0.0001	<0.0001
6	<0.0001	<0.0001
9	0.0001	0.0001
12	0.0013	0.0009
15	0.0142	0.0081
18	0.1018	0.0537
21	0.4237	0.2375
24	0.9702	0.6724
27	0.3914	0.7435
30	0.0997	0.2918

## Data Availability

The data presented in this study are available on motivated request from the corresponding author.
